# Raman spectroscopy, mobility size and radiative emissions data for soot formed at increasing temperature and equivalence ratio in flames hotter than conventional combustion applications

**DOI:** 10.1016/j.dib.2021.107064

**Published:** 2021-04-26

**Authors:** Shruthi Dasappa, Joaquin Camacho

**Affiliations:** a San Diego State University, San Diego California, USA; b University of California, San Diego, California, USA

**Keywords:** Soot, Carbon, Nanoparticle, Graphitization, Mobility sizing, Soot pyrometry, Raman spectroscopy

## Abstract

The dataset presented in this article is linked to the research article titled *“Evolution in size and structural order for incipient soot formed at flame temperatures greater than 2100 K”*[1]. The research article discusses the systematic evolution of flame formed carbon in premixed stagnation flames with flame temperatures hotter than conventional combustion applications. The effect of the growth environment on particle size, structure, composition and properties are studied. The flame temperature (1950 *K* < T_f,max_ < 2250 K) and equivalence ratio (Φ = 2.4, 2.5, and 2.6) are methodically varied to analyze impact on insipient soot while maintaining a comparable particle residence time (t_p_ ~ 15 ms). This article presents the data acquired for this systematic study. The data presented herein provides fundamental observations suitable for development of soot formation theory and modeling. Characterization of material properties and morphology are also relevant to potential applications of functional carbon nanomaterials. Raman spectra are measured for carbon films deposited from the flames, soot particle size distributions are obtained by aerosol sampling from the flames and soot radiative emissions are measured *in-situ* by color-ratio pyrometry. Deconvolution of Raman peaks is carried out to extract information on carbon bonding and structural order. Flame temperature is extracted from the measured color-ratio field making assumptions for the soot optical dispersion exponent.

**Specifications Table**

**Table:** 

Subject	Mechanical Engineering; Chemical Engineering
Specific subject area	Soot formation and carbon nanomaterials
Type of data	Table
How data were acquired	1. Radiative emissions of flame-formed carbon – Color-ratio pyrometry is employed using Nikon D3400 DSLR camera and bandpass filters (Andover FS20 - 450 nm, 650 nm and 900 nm) 2. Scanning mobility particle sizing – Particle size distribution is measured using TSI 1 nm Scanning Mobility Particle Sizer (TSI 3838E77) 3. Structure of flame-formed carbon – Off-line Raman spectroscopy is performed using a Thermo DXR2 Raman Microscope with a 532 nm excitation source
Data format	RawAnalyzed
Parameters for data collection	Premixed laminar stretch-stabilized flames with maximum flame temperatures spanning 1950 *K* < T_f,max_ < 2250 for equivalence ratio having Φ = 2.4, 2.5, and 2.6.
Description of data collection	1. Radiative emissions of flame-formed carbon – RAW images of the flames are captured using a Nikon DSLR (D3400) camera which are analyzed for color-ratio intensity to obtain the flame temperature and in-turn the dispersion exponent 2. Scanning mobility particle sizing – On-line aerosol sampling for mobility particle size distribution 3. Carbon bonding and structural order – Off-line Raman spectra for carbon films deposited from flames
Data source location	Department of Mechanical Engineering San Diego State University, San Diego, California, USA
Data accessibility	Data tables in Excel format submitted to Mendeley Data: https://doi.org/10.17632/yyxsgk3nm3.1
Related research article	S. Dasappa, J Camacho, “*Evolution in size and structural order for incipient soot formed at flame temperatures greater than 2100 K*”, Fuel, Volume 291, 1 May 2021, 120,196 https://doi.org/10.1016/j.fuel.2021.120196

## Value of the Data

• The data provide valuable insight into soot formation processes and material characterization for flame-formed carbon in the higher-temperature regime.
• Soot theory and modelers would benefit by using the data to evaluate model predictions for particle size distribution, carbon structure and optical properties for flame-formed carbon in the higher-temperature regime. Developers of applications for high-surface area carbon nanoparticles will also benefit from the systematic material characterization.
• The systematic approach shows clear trends with temperature and equivalence ratio which answer questions and open up new investigation pathways for soot formation and material properties of flame-formed carbon in the higher-temperature regime.

## 1 Data Description

The figures and dataset presented in this article is linked to the research article titled “*Evolution in size and structural order for incipient soot formed at flame temperatures greater than 2100 K*” [1]. The research paper systematically investigates the structure and particle size of soot formed in higher-temperature combustion applications. The effect of the growth environment on particle size, structure, composition and properties are studied. The flame temperature (1950 *K* < T_f,max_ < 2250 K) and equivalence ratio (Φ = 2.4, 2.5, and 2.6) are methodically varied to analyze impact on insipient soot while maintaining a comparable particle residence time (t_p_ ~ 15 ms). This article presents the data acquired for this systematic study.

### 1.1 Fig. 1

**Fig. 1:**
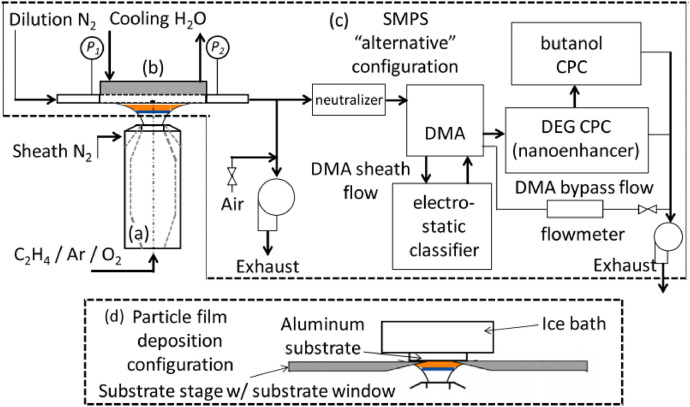
Experimental setup including the aerodynamic burner nozzle (a) a stagnation surface/sampling probe assembly (b) an SMPS in the “alternative” configuration defined by the vendor (c), and the setup for collecting flame formed carbon deposits for off-line analysis (d).

The Fig. 1 in this article shows the schematic description of the experimental setup including the aerodynamic burner nozzle, a stagnation surface/sampling probe assembly for on-line characterization, an SMPS in the “alternative” configuration and the setup for collecting flame formed carbon deposits for off-line analysis.

### 1.2 Fig. 2

**Fig. 2:**
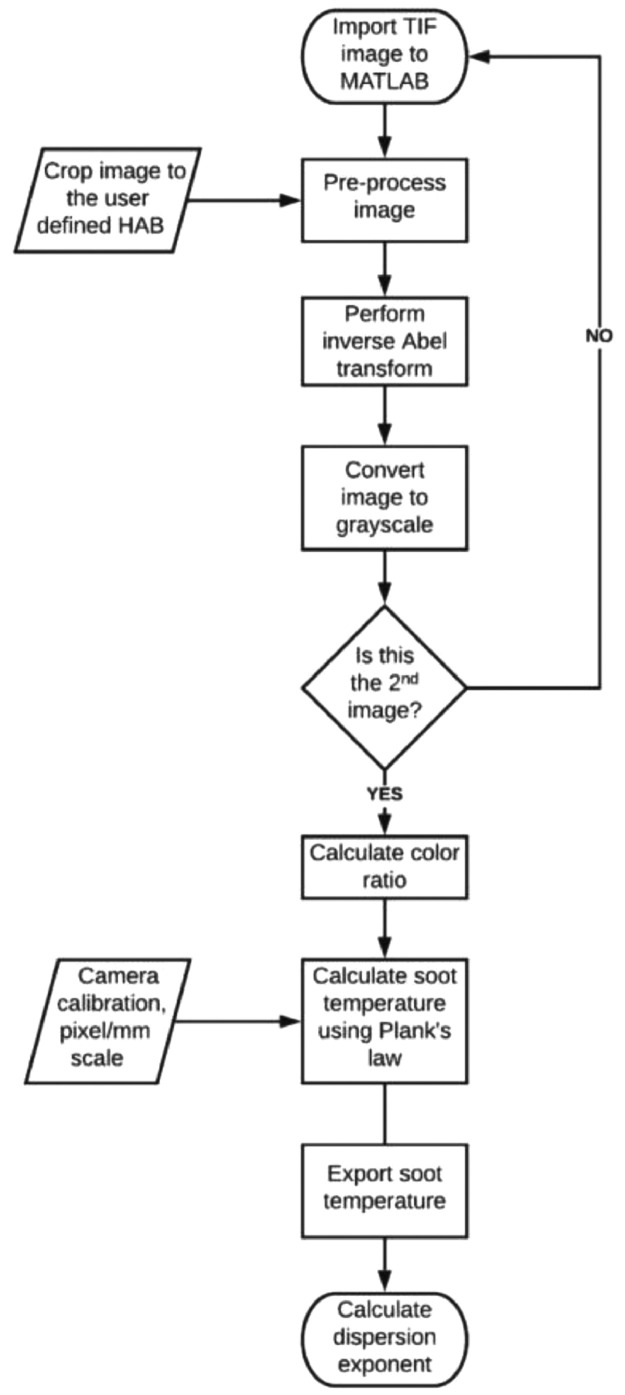
Typical workflow for color-ratio measurements and interpretation of radiative emissions.

The Fig. 2 in this article is the workflow that was adopted for the color-ratio measurements and calculation of the dispersion exponent for interpretation in terms of the radiative emissions.

### 1.3 Color ratio pyrometry

The spreadsheet “*Color_ratio_Data.xlsx*” available in the supplementary material contains multiple tables determining the 2-D flame temperature profiles for varying flame conditions. Sheets “*S1.1 − Φ = 2.4 T = 2190* *K CR*”,” *S2.1 − Φ = 2.4 T =* 2240 K *CR*”, “*S3.1. − Φ = 2.5 T =* 2155 K *CR*”, “*S4.1. − Φ = 2.5 T =* 2260 K *CR*”, “*S5.1. − Φ = 2.6 T =* 2135 K *CR*” and “*S6.1. − Φ = 2.6 T =* 2205 K *CR*” is the dataset of the measured color ratio fields for different flame conditions. Sheets “S1.2. *Φ =* 2.4 *T* = 2190K*”, “S2.2. Φ = 2.4 T = 2240K”, “S3.2 Φ = 2.5 T = 2155K”, “S4.2 Φ = 2.5 T = 2260K”, “S5.2. Φ = 2.6 T = 2135K”* and “*S6.2. Φ = 2.6 T = 2205K*” is the dataset of the flame temperature fields extracted assuming a particle optical dispersion exponent of unity for varying flame conditions. Sheet “*S7 Axial_centerline_profile*” is the dataset with the centerline axial temperature profiles with fitted dispersion exponent profiles based on computed axial temperature profiles for different flame conditions.

### 1.4 Scanning mobility particle sizing

The spreadsheet “*SMPS_Data.xlsx*” available in the supplementary material contains multiple tables for measured particle size distributions and global properties of the size distributions. Sheets “*S1.1 – Raw Φ = 2.4”, “S2.1 – Raw Φ = 2.5”* and *“S3.1 – Raw Φ = 2.6”* is the raw dataset of as-measured distributions before dilution correction, diffusion loss correction and adjustment of the mobility diameter for size-dependent transport behavior (denoted as Raw series). The sheets “*S1.2 – Corrected Φ = 2.4 ”, “S2.2 – Corrected Φ = 2.5”* and “*S3.2 – Corrected Φ = 2.6”* are the dataset of measured particle size distributions after the corrections. The correlations used for the correction are provided in the “*Table of contents*” sheet. The sheet “*S4 – Global Parameters*” is a dataset of the global parameters (distribution number density and volume fraction) of the size distributions for all flames studied.

### 1.5 Raman spectroscopy

The spreadsheet “*Raman_Spectra.xlsx*” available in the supplementary material contains the dataset with multiple tables for measured and analyzed Raman spectra for all flames studied. The sheet “*S1 Raw spectra*” is the raw data of as measured Raman spectra data for all flames studied. Sheet “*S2.1 – Primary peaks Φ = 2.4 ”, “S3.1 – Primary peaks Φ = 2.5*” and “*S4.1 – Primary peaks Φ = 2.6*” is the dataset with as measured Raman spectra, baseline corrected and the deconvoluted primary peaks of the spectra for all flames studied. The sheets “*S2.2 – Secondary peaks Φ = 2.4”, “S3.2 – Secondary peaks Φ = 2.5*” and “*S4.2 – Secondary peaks Φ = 2.6*” are the dataset with the as measured Raman spectra, baseline corrected and the deconvoluted secondary peaks of the spectra for all flames studied. The sheet “*S5 – Global Parameters*” contains the dataset providing the calculated global parameters for the Raman spectra for the primary bands along with the method used for the calculation.

## 2 Experimental Design, Materials and Methods

Premixed laminar stretch-stabilized stagnation flames are established with increasing flame temperature and equivalence ratio to observe the evolution in particle size distribution, carbon structure and optical properties of soot formed in the higher-temperature regime. These flames have a pseudo one-dimensional flame structure that can be manipulated to systematically observe carbon-formation in terms of fuel air ratio, growth time and temperature. The experimental setup, shown in Fig. 1, includes an aerodynamically shaped nozzle (D_nozzle_ = 1.43 cm) to induce a plug-flow at the inlet boundary and a water-cooled stagnation surface/ sampling probe assembly (nozzle-to-stagnation surface distance, *L* = 2.54 cm). Ethylene/oxygen/argon flames with a concentric shroud of nitrogen is employed for the study. The temperature at the nozzle and stagnation surface boundary are monitored by type K thermocouples with temperatures maintained at T_nozzle_ = 330 ± 20 K and T_stagnation_ = 400 ± 20 K. Calibrated critical orifices are used to control all gas flow rates. This configuration is similar to previous reports on soot formation in stretch-stabilized flames [2], [3], [4] and burner-stabilized stagnation flames [5], [6], [7], [8], [9], [10], [11], [12], [13], [14]. Deposition and analysis of metal oxide nanoparticles [15], [16], [17] was also studied using similar methods.

A methodical study is performed with 18 premixed stagnation flames with varying equivalence ratio (Φ = 2.4, 2.5 and 2.6) and flame temperature (1900 *K* < *T_f,_*_max_ < 2300 K) while maintaining a comparable particle residence time (*t_p_* ~ 15 ms). The flame structure computations carried out through OPPDIF in conjunction with USC Mech II [18], are used to design the flame series by determining maximum flame temperature and particle time as summarized in Table 1 and our reference publication [1].

**Table 1:** Detailed flame flow rates and parameters.

*X _C2H4_*	*X _O2_*	*X_Ar_*	*v_o_* (cm/s)	*T_f,_*_max_ (K)	*t_p_* (ms)	*a (1/s*)	*T _ad_ (K)*
*ϕ* = 2.4 series							
0.138	0.173	0.689	91	2035	18	36	1945
0.146	0.183	0.671	101	2085	15	40	2010
0.15	0.188	0.662	98	2110	15	39	2025
0.152	0.191	0.658	97	2140	15	38	2040
0.166	0.207	0.627	90	2190	14	35	2120
0.173	0.217	0.61	86	2240	14	34	2160
*ϕ* = 2.5 series							
0.145	0.174	0.681	104	1980	14	41	1905
0.151	0.18	0.67	87	2015	18	34	1950
0.160	0.192	0.648	90	2080	16	35	2005
0.175	0.21	0.615	101	2150	13	40	2075
0.183	0.22	0.597	97	2195	13	38	2110
0.196	0.236	0.569	88	2260	12	35	2180
*ϕ* = 2.6 series							
0.153	0.176	0.67	91	1950	18	36	1840
0.162	0.188	0.65	95	2050	16	37	1920
0.177	0.204	0.618	86	2080	16	34	1980
0.184	0.212	0.605	83	2110	16	33	2005
0.189	0.218	0.592	80	2135	16	31	2030
0.209	0.24	0.551	79	2205	14	31	2105

*T_f,_*_max_ computed using OPPDIF with measured boundary conditions: separation distance, *L* = 2.54 cm, *T_nozzle_* = 330 K and *T_stagnation_* = 400 K. *t_p_* is based on OPPDIF computed flame structure with thermophoretic velocity given by the hard sphere limit. *v_o_* is cold gas velocity, *a* is the global strain rate and *T_ad_* is the adiabatic flame temperature.

### 2.1 Color-ratio pyrometry

Color-ratio pyrometry is a valuable, non-intrusive measurement technique based on the incandescence nature of soot. Color-ratio pyrometry is carried out to analyze radiative emissions of the flame-formed carbon in-situ. A commercial DSLR camera (NIKON D3400) with the infrared filter removed was employed for capturing radiative emissions emanating from flame-formed carbon. Three separate images were recorded with different bandpass filters (Andover FS420, 450 nm, 650 nm and 900 nm) placed in front of the camera lens. Analysis is performed on an average of 10 frames. The camera captures the flame in a RAW format which is converted into TIF format using Nikon ViewNX-I. The images are further processed in MATLAB. The workflow used to compute the soot flame temperature from the captured flame images is shown in Fig. 2. The images are cropped to the user defined flame height above burner such that the axis of symmetry is in the center. The inverse Abel transform is used to translate the image into an axisymmetric radial profile. The color ratio of the greyscale translated images are used for soot temperature calculations.

The measurement technique proposed by Sunderland and co-workers [19] is adopted here with an additional modification to account for the evolution of optical properties as the soot structure evolves through the flame. The dispersion constant, α, accounts for the local properties of soot such as the H/C ratio which varies as the soot matures from a high H/C structure to a more carbonaceous structure. The wavelength dependent soot emissivity ε (λ) is proportional to λ^−α^ where λ is the filter wavelength and α is the light absorption dispersion exponent . The evolution in dispersion exponent was described by Gomez and co-workers [20,21] using this assumption. With the inclusion of α, color ratio could be interpreted in terms of Planck's Law as follows:

$$\frac{GS_{\lambda 1}}{GS_{\lambda 2}}=\frac{b_{1}\tau_{1}\Delta \lambda_{1}\lambda_{2}^{5+\alpha }exp\left(\frac{hc}{\lambda_{2}kT}\right)}{b_{2}\tau_{2}\Delta \lambda_{2}\lambda_{1}^{5+\alpha }exp\left(\frac{hc}{\lambda_{1}kT}\right)}$$

where h is the Planck's constant, k is the Boltzmann's constant, T is the temperature of the particle (assumed to be equal to the surrounding gas) and $\lambda_{i}$ is the rated wavelength of the bandpass filter. $GS_{i}\left(r\right)$ is the post Abel transform radial greyscale output from the camera sensor through the respective filter and $b_{i}$ is the wavelength specific fitting constant relating the camera response to spectral intensity through the Planck's law, $\tau_{i}$ is the transmissivity of the filter and $\Delta \lambda_{i}$ is the FWHM of the filter. $b_{i}$ is calibrated using a blackbody furnace (Newport Oriel 67,032) with temperatures ranging from 1173 K to 1473 K while confirming the linear response of the camera. The temperature contours reported correspond to α = 1 which is typical of mature soot [19,22]. α is further evaluated as a function of local radiative properties of flame formed tuned to agree with the well-established temperature profile of OPPDIF. This along with the corrected axial centerline temperature profile is presented in the article.

### 2.2 Mobility particle sizing

Mobility particle sizing for the soot PSDF measurement is carried out by aerosol sampling at the stagnation surface. An 8 cm diameter by 1.3 cm thick aluminum disk acts as the flow stagnation surface with a 0.635 cm stainless steel tubular sampling probe embedded into the disk. The tube wall is from embedded flush with the stagnation surface and parallel to the flat flame. The flame sample was drawn into the probe through a laser-drilled micro-orifice (*D_orifice_* = 130 µm). The orifice was positioned on the center axis of the burner facing the incoming flame gas. Established aerosol sampling techniques are applied here to minimize loss of the smallest particles due to diffusion and artificial probe-induced coagulation [4,^5^,10]. The optimum dilution applied is 2000 for all measure particle size distributions reported. A TSI 1 nm Scanning Mobility Particle Sizer is used here (TSI 3838E77) for particle sizing by differential mobility analysis. This system is composed of a dual voltage classifier (TSI 308,202), a Kr-85 bi-polar diffusion charger (Neutralizer TSI 3077A), 1 nm differential mobility analyzer (DMA) (TSI 3086), a diethylene glycol-based (DEG) condensation particle counter (CPC) (so-called Nanoenhancer, TSI 3777) and a butanol-based CPC (TSI 3772). TSI Aerosol Instrument Manager Software (version 10.2) is used to collect and export the PSDFs. The DEG-based CPC is meant to allow for counting of particles with mobility diameters as small as 1.4 nm. Mobility sizing of soot in the DMA can classify particles approaching 1 nm but counting particles in this size range has required faraday cup electrometers DEG-based CPCs.

The SMPS is in the “compact” configuration suggested by TSI to minimize diffusion losses before the CPCs. The flow into the neutralizer is 5 SLPM and 2.5 SLPM flows into the DMA, the balance of which bypasses to exhaust. The sheath flow through the DMA (25 SLPM) is controlled in the electrostatic classifier housing and this gives a 10:1 sheath to aerosol flow. The DMA polarity is set to positive in the current work. TSI Aerosol Instrument Manager Software (version 10.2) is used to collect and export the measured PSDFs. An insert supplied by the vendor is mounted onto the inlet of the neutralizer to minimize flow recirculation for more predictable attainment of the equilibrium charge distribution. TSI also recently measured the penetration of ultra-fine particles through the system flow path and a new diffusion correction is applied to the measured PSDF. The penetration factor is presented in this article. The SMPS mobility diameter was corrected to yield the real diameter on the basis of nanoparticle transport theory. The correlation between the SMPS measured mobility diameter *D_p,SMPS_* and true diameter *D_p_* of a carbonaceous particle is given by [23].

$$\frac{D_{P}}{D_{P,SMPS}}=\tanh\left(1.4334+0.01248\;D_{P,SMPS}\right)\;\left(1.0676\;-\;\frac{0.4463}{D_{P,SMPS}}\right)$$

The data as obtained by the SMPS along with a log-normal curve fitting it presented here. The curve fitting parameters are also studied to provide insight into the competing soot formation processes in terms of global properties of the size distribution.

### 2.3 Raman spectroscopy

Raman spectroscopy was carried out off-line for flame formed carbon collected from each flame of the 18 flames examined. A stagnation surface, shown in Fig. 1, is designed to serve as a stage for deposition substrates over the flame. The stage is a 2 mm thick aluminum, water-cooled stagnation surface that was designed with a 3 cm diameter window to mount 1 mm thick aluminum plates over the area of the flat flame. The window was tapered such that deposition plates were effectively flush giving a nozzle-to-stagnation separation distance identical to the corresponding probe sampling experiments. Aluminum was chosen over quartz as the deposition surface material because high thermal conductivity is required to maintain the 400 K temperature at the deposition surface. An ice bath is further used to cool the water flowing through the heat exchanger. The temperature of the deposition surface was monitored by a thin wire (0.125 µm) type K thermocouple placed on the non-deposition side of the aluminum plate. The deposition temperature was maintained at 430 ± 20 K for all flame conditions. A Thermo DXR2 Raman Microscope with a 532 nm excitation source was used to measure Raman spectra with Raman shifts spanning 100 – 3500 cm^−1^. The laser was focused under a 50x objective at 1 mW power and care was taken to prevent modifications to the carbon structure by laser excitation.

The measured spectra are analyzed for first order spectra (Raman shift: 1000 – 1800 cm^−1^) and second order spectra (Raman shift: 1800 – 3500 cm^−1^). Spectrum intensities are normalized to the highest intensity value measured in the spectrum and a linear correction was used in MATLAB to correct the luminescent background. A five peak deconvolution is performed on the measured first order Raman spectra to examine the structure of flame formed carbon. The curve fitting included 4 Lorentzian curves (G, D, D’, D4) and a Gaussian curve (D3). A four peak (2D_1_, 2D_2_, *D* + *G* and 2D’) deconvolution is performed on the second order Raman spectra using Lorentzian curve fitting. The peak positions of the curves were moderately constrained while the other parameters were allowed to vary to match the measured spectra.

The data presented in this article consists of the raw Raman spectra, background corrected Raman spectra and deconvoluted Raman spectra for the flames conditions presented in Table 1. Global parameters like full width at half maximum (FWHM), defect distance and defect density are also studied to provide insight into the structure of flame formed carbon.

## Ethics Statement

Not applicable.

## CRediT Author Statement

**Shruthi Dasappa:** Conceptualization, Methodology, Formal analysis, Writing - review & Editing; **Joaquin Camacho:** Conceptualization, Methodology, Formal analysis, Writing - review & Editing.

## Declaration of competing interest

The authors declare that they have no known competing financial interests or personal relationships which have, or could be perceived to have, influenced the work reported in this article.
